# Reciprocity-induced symmetry in the round-trip transmission through complex systems

**DOI:** 10.1063/5.0021285

**Published:** 2020-10-07

**Authors:** Szu-Yu Lee, Vicente J. Parot, Brett E. Bouma, Martin Villiger

**Affiliations:** 1 Harvard Medical School and Massachusetts General Hospital, Wellman Center for Photomedicine, Boston, Massachusetts 02114, USA; 2 Harvard-MIT Health Sciences and Technology, Massachusetts Institute of Technology, Cambridge, Massachusetts 02139, USA; 3 Institute for Medical Engineering and Science, Massachusetts Institute of Technology, Cambridge, Massachusetts 02139, USA

## Abstract

Reciprocity is a fundamental principle of wave physics and directly relates to the symmetry in the transmission through a system when interchanging the input and output. The coherent transmission matrix (TM) is a convenient method to characterize wave transmission through general media. Here, we demonstrate the optical reciprocal nature of complex media by exploring their TM properties. We measured phase-corrected TMs of forward and round-trip propagation in a single polarization state through a looped 1 m-long step-index optical multimode fiber (MMF) to experimentally verify a transpose relationship between the forward and backward transmission. This symmetry impedes straightforward MMF calibration from proximal measurements of the round-trip TM. Furthermore, we show how focusing through the MMF with digital optical phase conjugation is compromised by system loss since time reversibility relies on power conservation. These insights may inform the development of new imaging techniques through complex media and coherent control of waves in photonic systems.

## 1. INTRODUCTION

The bi-directional transmission through photonic systems is governed by the universal Lorentz reciprocity (or the Helmholtz reciprocity), which states that light propagating along a reversed path experiences the exact same transmission coefficient as in the forward direction, independent of the path complexity^1,2^ or the presence of loss.^3–5^ In the linear regime, this suggests a definite relation, or symmetry, between the forward and the backward transmission when interchanging the source and detector. This symmetry not only underlies the behavior of common optical components, such as polarizers, beam splitters, and wave-plates, but also engenders surprising physical phenomena in complex systems such as coherent backscattering (or weak localization) and Anderson localization.^6,7^ Optical phase conjugation is a well-known consequence of this symmetry in loss-free systems, whereby an original light distribution is replicated by reversing the propagation direction of the detected field while conjugating its wave-front. Digital optical phase conjugation (DOPC) has been well established for focusing and imaging through complex or disordered media, including multimode fibers (MMFs).^8–11^ However, the more general underlying transmission symmetry of bi-directional light transmission through complex systems and its implications have not been explicitly demonstrated and discussed.

Light transmission through MMFs is typically considered a chaotic process, as the modal scrambling results in the generation of random speckle patterns at the output.^12^ MMFs are of particular interest for studying optical transport in complex media due to their finite degrees of freedom (DOFs) and optical energy confinement. Owing to their high data transmission capacity in an ultra-small footprint, MMFs have gained significant attention and hold great promise for optical communication and biomedical endoscopic applications.^13,14^ For instance, once measured, the apparently chaotic transmission through a MMF can be harnessed to relay image information from the distal to the proximal fiber end, enabling the reconstruction of an image of the distal object.^15–17^ Nevertheless, imaging through MMFs remains technically challenging and exploring fundamental properties of MMF light transmission may help develop new strategies to advance MMF-based imaging and endoscopy.^18^

In the present study, we investigate MMF transmission properties using a monochromatic coherent transmission matrix (TM) formalism^19,20^ and experimentally demonstrate the transpose symmetry between the forward and backward TMs in this complex medium imposed by general optical reciprocity. The TM description is a subpart of the common scattering matrix formalism^1,19,21^ and offers a simpler framework that decouples the input and output channels. We also show that while DOPC enables focusing through MMFs, the focusing performance declines with increasing loss since the time reversibility is corrupted when power is not conserved. Finally, we discuss the implications of the resulting transpose symmetry on calibrating MMF transmission with only access to the proximal side, which is critical for practical MMF imaging. The gained insights are readily applicable to general electromagnetic transport in complex and disordered media.

## II. RESULTS

### A. Measuring the single-pass forward T_fw_ and double-pass round-trip T_2X_ of a MMF

**FIG. 1.**
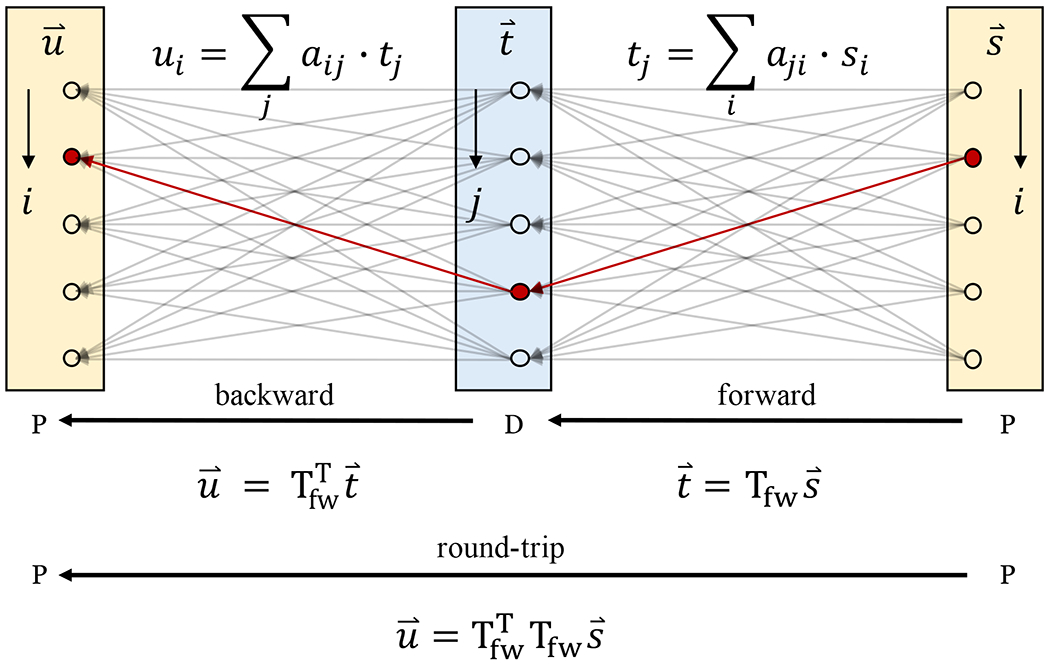
Schematic of forward and backward TMs characterizing transmission between the proximal (P) and distal (D) ends of a linear optical system. The round- trip transmission from and to the proximal end is unfolded to reveal the hidden transpose symmetry when flipping the direction of an optical path (gray arrows) linking a pair of spatial channels. The vectors $\vec{u}$, $\vec{s}$, and $\vec{t}$ represent complex fields with constituent spatial channels indexed by *i* and *j* on the proximal and distal ends, respectively. Each element *a_ji_* of the forward TM describes the complex contribution of proximal input channel *i* to distal output channel *j*. The red arrows link a pair of spatial channels in the forward and backward transmission. Owing to reciprocity, both directions feature the same transmission coefficient, yet they correspond to transposed elements in the corresponding TMs, with interchanged row and column indices.

As shown in Fig. 1, the optical transmission through a general medium from an input surface to an output surface can be expressed by a TM, where each element is a complex coefficient specifying the amplitude and phase evolution of the transmitted monochromatic field between the corresponding pair of input and output spatial channels. The spatial channels correspond to the sampling locations on the input and output surfaces, respectively, and are assumed to be sufficiently dense to correctly sample the electromagnetic fields. We can then express forward light transmission, T_fw_, from the proximal to distal end as

(1) $$\vec{t}={\text{T}}_{\text{fw}}\vec{s},$$

where $\vec{t}$ and $\vec{s}$ are the vectorized representations of the distal output field and proximal input field, respectively. If $\vec{t}$ and $\vec{s}$ are ordered first by the spatial modes, and then by polarization, T_fw_ can be partitioned into four blocks,

(2) $${\text{T}}_{\text{fw}}=[\begin{array}{cc} {\text{T}}_{\text{XH}} & {\text{T}}_{\text{XV}}\\ {\text{T}}_{\text{YH}} & {\text{T}}_{\text{YV}} \end{array}],$$

where the subscripts X, Y and H, V denote two orthogonal polarization states on the distal and proximal side, respectively. The backward light transmission from the distal to proximal end can be written as

(3) $$\vec{u}={\text{T}}_{\text{bw}}\vec{t},$$

where $\vec{u}$ and $\vec{t}$ are the proximal output field and distal input field, respectively. According to the reciprocity theorem, light propagating along the reversed path between the input and output will experience the same transmission coefficient as in the forward direction. In the context of Jones matrices, which describe the relation between the polarization states of the input and the output field propagating through an optical system, de Hoop’s notion of reciprocity manifests as a transpose relationship between the Jones matrices describing forward and reverse transmissions.^2^ By analogy with the Jones matrix formalism, when interchanging the input and output spatial channels of the medium, reciprocity instructs that T_bw_ is the transpose of T_fw_,

(4) $${\text{T}}_{\text{bw}}={\text{T}}_{\text{fw}}^{\text{T}}=[\begin{array}{cc} {\text{T}}_{\text{XH}}^{\text{T}} & {\text{T}}_{\text{YH}}^{\text{T}}\\ {\text{T}}_{\text{XV}}^{\text{T}} & {\text{T}}_{\text{YV}}^{\text{T}} \end{array}],$$

where the superscript T indicates the regular matrix transpose. In addition, since sequential light transmission is modeled as TM multiplication, the round-trip transmission through the same medium, T_2X_ (light transmits to and is reflected from the distal side; then, it travels back to the proximal side), equals the product of T_bw_ and T_fw_,

(5) $${\text{T}}_{2\text{X}}={\text{T}}_{\text{bw}}{\text{T}}_{\text{fw}}={\text{T}}_{\text{fw}}^{\text{T}}{\text{T}}_{\text{fw}},$$

making T_2X_ a transpose symmetric matrix,

(6) $${\text{T}}_{2\text{X}}=\left[\begin{array}{cc} {\text{T}}_{\text{XH}}^{\text{T}}{\text{T}}_{\text{XH}}+{\text{T}}_{\text{YH}}^{\text{T}}{\text{T}}_{\text{YH}} & {\text{T}}_{\text{XH}}^{\text{T}}{\text{T}}_{\text{XV}}+{\text{T}}_{\text{YH}}^{\text{T}}{\text{T}}_{\text{YV}}\\ {\text{T}}_{\text{XV}}^{\text{T}}{\text{T}}_{\text{XH}}+{\text{T}}_{\text{YV}}^{\text{T}}{\text{T}}_{\text{YH}} & {\text{T}}_{\text{XV}}^{\text{T}}{\text{T}}_{\text{XV}}+{\text{T}}_{\text{YV}}^{\text{T}}{\text{T}}_{\text{YV}} \end{array}\right]={\text{T}}_{2\text{X}}^{\text{T}}.$$

Of note, the two on-diagonal blocks are self-transpose-symmetric and the two off-diagonal blocks are instead the transpose of each other.

**FIG. 2.**
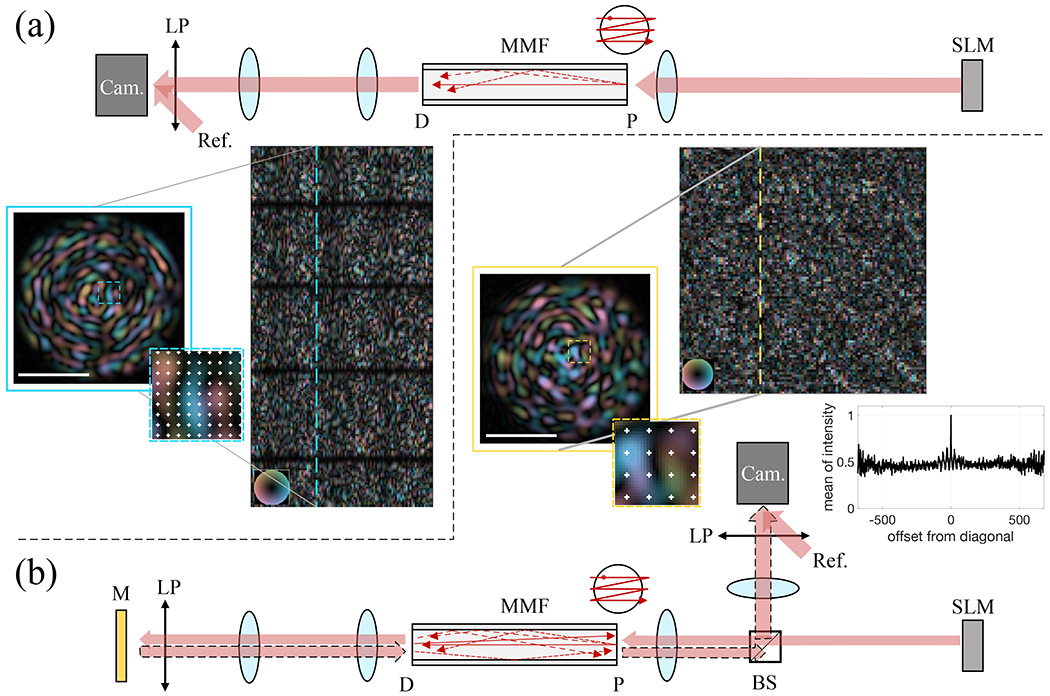
Measurements of the MMF TMs. The fiber, although drawn as if it were straight, was in fact coiled in experiments. P: proximal, D: distal, LP: linear polarizer, Ref.: reference wave, Cam.: camera, M: gold-coated mirror, and BS: beam splitter. (a) A focus was scanned by using the SLM across 695 positions distributed over the MMF proximal facet. The output light field interfered with a reference wave on the camera and created a modulated image, which could be processed through Hilbert transformation into the complex amplitude of the output speckle. The image was down-sampled, as exemplified in the magnified inset, and rearranged into a column vector of T_fw_ with rows and columns indexed by the output and input channel positions, respectively. Only a subset of T_fw_ is shown. (b) For round-trip measurements, the camera at the distal side was replaced by a mirror, and the returning light was directed by using a non-polarizing beam splitter to the same camera for holographic recording. The complex image of the round-trip output speckle was down-sampled at the 695 positions of the input foci grid (inset), resulting in a square matrix. A subset of T_2X_ is shown, the vertical dashed line indicates the vector arranged as an image of the facet, and the yellow inset shows sampling locations as white markers. The color maps encode complex values, and the scale bars in the insets are 50 *μ*m. The plotted trace is the average intensity of matrix elements with varying offset from the diagonal, and the ratio of two between the main diagonal and the off-diagonal reveals the coherent backscattering in the MMF.

To experimentally verify Eqs. (4) and (5), we measured the monochromatic TMs, T_fw_ and T_2X_, of a 1-m-long MMF randomly coiled with a minimum radius of curvature of 23 mm, using the setup shown in Fig. 2. A laser beam (λ = 1550 nm and linewidth < 100 kHz) was linearly polarized in a vertical (V) polarization state, reflected on a phase-only spatial light modulator (SLM, Model P1920-850-1650-HDMI, Meadowlark Optics) in the same polarization state, and then focused by using an objective lens (Mitutoyo Plan Apo NIR Infinity Corrected) with a numerical aperture (NA) of 0.4 into a 2.5 *μ*m full-width at half maximum (FWHM) spot on the facet of the step-index MMF with 105 *μ*m core diameter and a NA of 0.22 (FG105LCA, Thorlabs). An offset phase ramp was applied to the SLM to block unmodulated light from entering the fiber. The MMF theoretically supports ~550 guided modes per linear polarization.^22^ The angular spectrum of the spot exceeded the NA of the MMF to ensure efficient population of high-order modes. To measure T_fw_, the forward TM [Fig. 2(a)], we coupled the focal spot into the MMF through input spatial channels on the proximal side and imaged the speckle pattern exiting from output spatial channels on the distal side with another identical objective and a tube lens (f = 30 cm) onto an InGaAs camera (OW1.7-VS-CL-LP-640, Raptor Photonics) with a vertically oriented, linear polarizer (LP) placed in front of it. A tilted plane reference wave, polarized by the same polarizer, interfered with the speckle pattern to record the complex image of the speckle pattern field through off-axis holography in the V polarization state. If we consider the V polarizers at the proximal and distal sides as part of the system whose TM we are measuring, then T_YV_ = T_VV_ and T_fw_ becomes

(7) $${\text{T}}_{\text{fw}}=[\begin{array}{cc} 0 & 0\\ 0 & {\text{T}}_{\text{VV}} \end{array}].$$

To release digital storage burden, we down-sampled the complex image at a defined grid of 2637 positions. To uniformly probe all MMF guided modes, this procedure was repeated in an oversampling fashion for a dense grid of 695 equally spaced illuminating foci sequentially generated by phase gradients on the SLM. Rearranging column by column the ensemble of vectorized complex output images recorded over all input spatial channels constructed T_fw_ representing the linear transformation of light traveling from the proximal facet to the distal facet. Due to the difference in the number of input and output sampling positions, T_fw_ is a tall rectangular matrix.

For measuring T_2X_, the double-pass TM, as shown in Fig. 2(b), we again sequentially coupled light into the MMF from the proximal end through the same set of input spatial channels. On the distal side, we replaced the camera used for measuring in the forward transmission with a gold-coated mirror to reflect the light back into the MMF. The same V linear polarizer, previously in front of the camera and now in front of the gold-coated mirror, was necessary to maintain the identical T_fw_ and avoid polarization crosstalk. In general, the spatial and polarization DOFs are coupled through mode mixing during light propagation in the MMF, and the MMF output polarization states are different from the input polarization state.^23^ With the distal and proximal V linear polarizers, we measure the transmission from a V linear input polarization state into a V linear output polarization state, both for the forward and the double-pass TMs. T_2X_ of Eq. (6) simplifies in this case to

(8) $${\text{T}}_{2\text{X}}=[\begin{array}{cc} 0 & 0\\ 0 & {\text{T}}_{\text{VV}}^{\text{T}}{\text{T}}_{\text{VV}} \end{array}].$$

On the proximal side, we recorded the round-trip transmission by decoupling its path from the illumination with a non-polarizing beam splitter. To preserve the symmetry between the illumination and the detection configurations and to obtain a square matrix T_2X_, we sampled the recorded output fields at the 695 positions defined by the input focus positions. Furthermore, to mitigate specular reflections at both the distal and proximal facets, wedge prism mounting shims (SM1W1122, Thorlabs) filled with index-matching gel (G608N3, Thorlabs) were used to cover both facets for measurements of forward and double-pass TMs. Intriguingly, the round-trip measurements through individual proximal spatial channels allow us to observe the coherent backscattering effect, which guarantees constructive interference in pairs of time-reversed optical paths, and thus, light is statistically twice as likely to exit through the same spatial channel that it used to couple into the fiber than through any other output channel.^6^ In the TM formalism, this corresponds to a ratio of two between the mean intensities of the main diagonal and off-diagonal elements in T_2X_, as plotted in Fig. 2(b). Mathematically, if we assume that the elements in T_fw_ feature independent real and imaginary parts following identical normal distributions, then Eq. (5) states that T_2X_ is the same as a pseudo-covariance matrix (or relation matrix) of proper complex random vectors,^24,25^ resulting in the factor of two due to Gaussian statistics.

In our experiments, we used a single polarization for illumination and detection to avoid the experimental complexity of measuring polarization-resolved TMs.^26^ Furthermore, the X and Y polarization states at the distal side were identical to the H and V polarizations at the proximal side. Measuring the round-trip TM without the distal V polarizer would still result in a transpose symmetric matrix T_2X_, but the coupling between the polarization states would create a second term ${\text{T}}_{\text{HV}}^{\text{T}}{\text{T}}_{\text{HV}}$. Hence, the distal V polarizer was required when measuring the round-trip TM to be able to relate T_2X_ to the measured T_VV_ of T_fw_. The polarization degree of freedom simply extends the DOFs of the spatial modes, and in analogy to the partition of the transmission matrix into the four polarization blocks of Eq. (5), we could also partition T_VV_ into any two subsets of input and output spatial modes. Thus, T_VV_ can likewise be defined as being composed of four blocks, which express the transmissions from the two input subsets to the two output subsets. The measured round-trip matrix ${\text{T}}_{\text{VV}}^{\text{T}}{\text{T}}_{\text{VV}}$ contains two on-diagonal blocks that are self-transpose-symmetric and two off-diagonal blocks that are the transpose of each other. By extension, the experimental verification of the symmetry relation for a single polarization state holds for any combination of spatial channels and polarization states and holds without loss of generality.

### B. Inspecting and controlling the spatial degrees of freedom

**FIG. 3.**
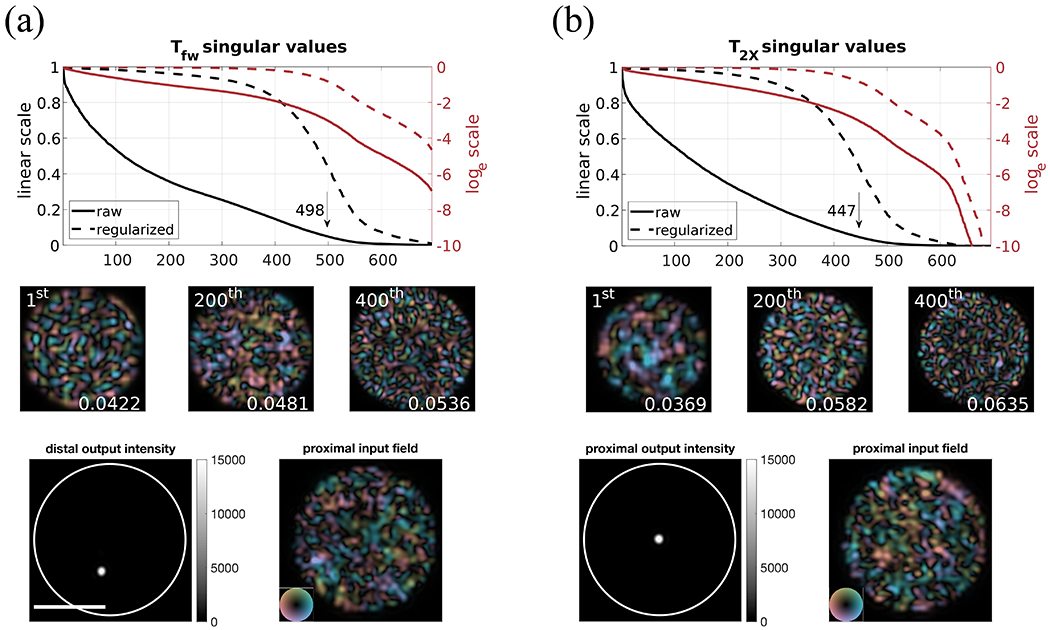
Singular values (SVs) of the measured TMs and focusing through the MMF with regularized inversions in (a) single-pass forward T_fw_ and (b) double-pass round-trip T_2X_. The black arrows indicate the number of modes with an SV above 5% of the TM’s largest SV. The solid and dashed curves correspond to raw and regularized SVs, while black and red lines show linear and log scales, respectively. Three examples of singular modes are visualized for each configuration by reshaping singular vectors to 2D images and numerically interpolating the images for better visual appearance. The averaged normalized radial frequency (0.5 cycles/radius) of the power spectral density of each mode is indicated in the lower-right corner. High-order modes are associated with higher radial frequency and are subject to increased loss. As shown in the bottom row, with the knowledge of T_fw_ and T_2X_, we can focus through the MMF on either the distal or the proximal facet at an intended position by tailoring the illumination wave-front at the proximal end. The white circles outline the fiber facet, and the scale bar is 50 *μ*m.

We quantified the number of guided modes within the MMF by performing singular value decomposition (SVD) on measured T_fw_ and T_2X_, counting the singular values (SVs) above a threshold defined as 5% of the largest SV. As shown in Fig. 3, there are ~500 populated modes in T_fw_ and ~450 in T_2X_, but mode-dependent transmission loss is apparent. While the numbers are consistent with a theoretical maximum of 550, when inspecting the left singular vectors associated with decaying SVs, we find that the loss of guided power increases as a mode carries higher radial frequencies. T_2x_ exhibits more severe loss due to the double passage through the MMF. We attribute these losses to coupling and detection of a single polarization state and to oversampling and interpolation of TM measurements.

To show that the measured TMs are accurate, we controlled the amplitude and phase of the illumination wave-front to physically create a sharp optical focus through the MMF on either the distal or the proximal end, using the measured T_fw_ and T_2X_, respectively. This was accomplished by numerically inverting the TMs and generating the required phase and amplitude pattern at the input pupil of the system for an intended focusing position at the output. The required patterns were generated with a phase-only SLM relayed through a 4f system to the input pupil, using the Gerchberg–Saxton (GS) algorithm to determine a suitable phase pattern.^27^ Because T_fw_ is non-square, and both matrices are corrupted by noise and close to singular, we approximated matrix inversion with Tikhonov regularization, T^−1(tik)^, with the regularization parameter, *γ*, chosen as 10% of the greatest SV. This is justified based on the L-curve method.^28^ The product of T_fw_ with its regularized inverse is identical to the multiplication of a modified TM with its Hermitian transpose. The modification consists of rescaling each SV, *σ*, of the TM by $1/\sqrt{\sigma^{2}+\gamma^{2}}$ and is shown in dashed curves (labeled as “regularized”) in Fig. 3. Examples of the V polarization of the created foci are shown in the bottom row of Fig. 3, with ~4 *μ*m average FWHM. We defined a focus contrast (FC) as the ratio of the peak intensity at the focal point over the average intensity across all output spatial channels to evaluate the focusing performance. This FC metric is similar to the enhancement factor defined by Vellekoop *et a1.*,^29,30^ but it is bounded by the number of guided modes in the MMF even under a lossless condition and expresses accurately what fraction of the DOFs is effectively controlled. The average FCs for distal and proximal focusing were ~205.7 and ~148.3, respectively. Despite the fact that the maximal FC, calculated when assuming the total power is concentrated in a single output spatial channel, would be 550 given the theoretical number of modes per polarization, the experimental FCs are limited by several factors such as the MMF loss, the finite persistence time of the system, the measurement noise, the imperfect wave-front shaping, and the finite camera dynamic range. Despite the discrepancy between the experimental and theoretical values, the achieved FCs agree with the quantified number of modes, suggesting that we were reasonably exploiting the available DOFs.

### C. Transpose symmetry in round-trip transmission

**FIG. 4.**
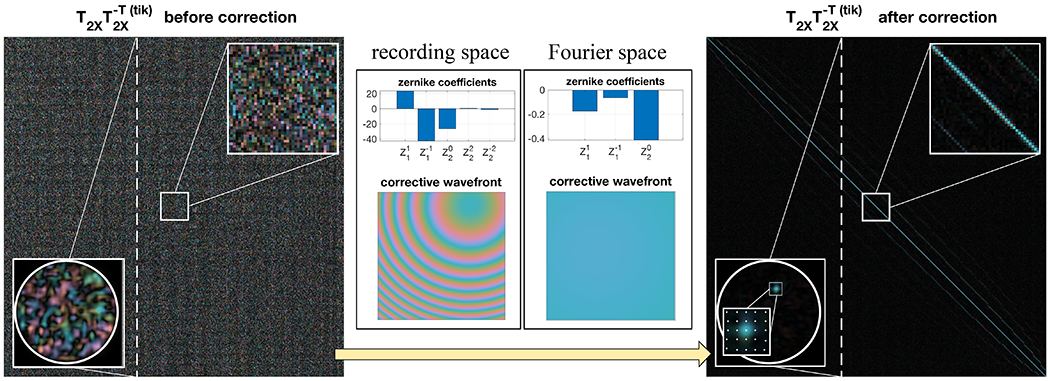
Transpose symmetry of T_2X_ before and after correction for misalignment at the proximal end. The phase mismatch and positional shifts are described as Zernike polynomials in the recording and Fourier space, respectively, and the amplitude of each mode is iteratively updated to minimize the difference between T’_2X_ and ${\text{T}}_{2\text{X}}^{,\text{T}}$. Higher order Zernike polynomials are found to be negligible after additional trials. Using a Newtonian-based optimizer, the normalized error converges from 200% to 23% after the phase mismatch and positional shift corrections, each within tens of iterations. The horizontal and vertical tilts and defocus in the recording space are the dominant factors. A column in the products of T_2X_ and ${\text{T}}_{2\text{X}}^{-\text{T}\left(\text{tik}\right)}$ before and after phase correction is selected, converted back into 2D coordinates, and smoothed by interpolation to illustrate constructive interference at the corresponding proximal spatial channel when using corrected TMs. The offset diagonals on both sides of the main diagonal are due to oversampling during TM measurement, as visualized by indicating the proximal sampling positions.

Next, in a subsequent experiment, we set out to verify the anticipated transpose symmetry within the round-trip TM T_2X_, as stated in Eq. (6). This property should be self-sustained and independent of T_fw_. Physical misalignment between the defined input surface to the MMF and the image recording plane at the proximal end introduces a phase mismatch and relative shifts that need to be compensated to reveal the underlying transpose symmetry. This is similar to misalignment issues in common DOPC systems.^31^ We parameterized the physical misalignment considering 8 variables and developed an optimization procedure that corrects the alignment imperfections, following Plöschner *et al.*^26^ To address the phase mismatch, we applied a two-dimensional (2D) phase term constituted by Zernike polynomials in the recording space of the output spatial channels. This corresponds to a diagonal phase-only matrix left-multiplied with T_2X_. The Zernike orders correspond to 2D tilts, defocus, and 2D astigmatisms. To register the positional shifts, we applied another phase term with 2D tilts and defocus in the Fourier space of the output spatial channels of T_2X_, as this is the same as the lateral and axial translation of the observation coordinates. This correction is equivalent to convolving the output spatial channels with a complex and offset point spread function. In the TM formalism, this is a further left-multiplication of T_2X_ with a Toeplitz matrix. The Zernike coefficients were determined by minimizing the error ${\left|{\text{T}}_{2\text{X}}^{’\text{T}}-{\text{T}}_{2\text{X}}^{’}\right|}^{2}$, where T’_2X_ is the corrected T_2X_ and |·|^2^ is the squared Frobenius matrix norm. Without correction, the initial error, normalized by |T_2X_|^2^, was 200%. With correction, the normalized error was reduced to 23%. For comparison, we found a 15% residual error when computing the normalized squared Frobenius norm of the difference between two sequentially measured round-trip TMs of the identical MMF transmission. To investigate transpose symmetry, we verified the diagonal localization in the product of the matrix by its inverse transpose. The product of the uncorrected T_2X_ with its Tikhonov regularized transpose matrix inversion ${\text{T}}_{2\text{X}}^{-\text{T}\left(\text{tik}\right)}$ is a chaotic matrix due to the disordered interference between populated modes caused by the physical misalignment (Fig. 4). However, after applying the correction, the product of T’_2X_ with ${\text{T}}_{2\text{X}}^{’-\text{T}\left(\text{tik}\right)}$ became close to the identity matrix, with the integrated on-diagonal energy over the total matrix energy improving from 0.24% to 43.5%. As benchmark, the same metric applied to a perfectly symmetric TM, $\left({\text{T}’}_{2\text{X}}^{\text{T}}+{\text{T}’}_{2\text{X}}\right)/\text{2}$, resulted in 59.2% on-diagonal energy, limited by the regularized matrix inversion. These results show that the phase-corrected round-trip TM matches its transpose, thus demonstrating its transpose symmetry.

### D. Transpose relationship between the backward and forward transmission

**FIG. 5.**
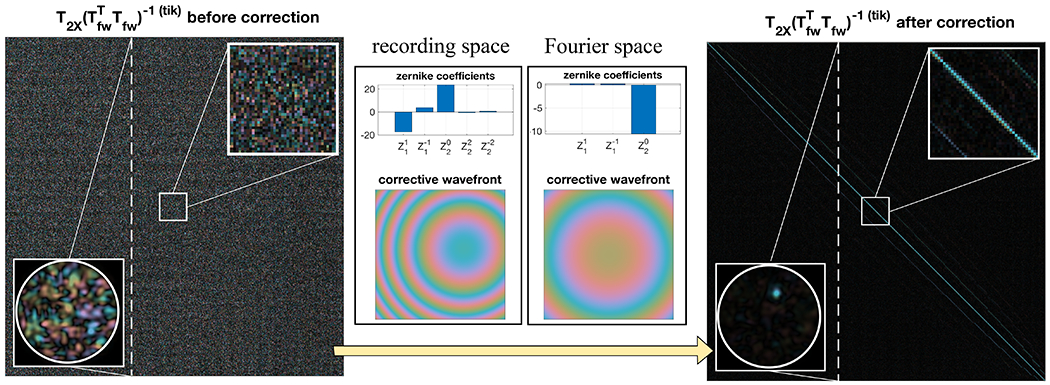
Visualization of optical reciprocity within the MMF after correcting f for misalignment. During optimization, the amplitude of each Zernike polynomial is iteratively updated to minimize the difference between experimental and synthesized round-trip transmission. The Newtonian-based optimizer was again used to find the optimal correction. The on-diagonal energy ratio improved from 0.27% to 36.6% after the phase mismatch and positional shift corrections, each within tens of iterations.

With the corrected T_2X_, we proceeded to verify the transpose relationship between the forward and backward TMs, as stated in Eq. (4). For experimental convenience, instead of directly comparing T_bw_ and T_fw_, we assumed that ${\text{T}}_{\text{bw}}={\text{T}}_{\text{fw}}^{\text{T}}$ and worked with ${\text{T}}_{\text{fw}}^{\text{T}}{\text{T}}_{\text{fw}}$ and T’_2X_, avoiding the complexity of directly measuring T_bw_. Similar to correcting the round-trip measurements, we had to compensate the physical misalignment between the recording plane on the distal side for measuring T_fw_ and the gold-coated mirror used in measuring T_2X_. In a similar way to how we corrected T_2X_, we applied phase terms to the recording and Fourier spaces of the output spatial channels of T_fw_. In this case, we aimed to minimize the error ${\left|{\text{T}’}_{2\text{X}}-{\text{T}’}_{\text{fw}}^{\text{T}}{\text{T}’}_{\text{fW}}\right|}^{\text{2}}$, where T’_fw_ is the corrected T_fw_. Figure 5 shows that the misalignment, characterized by the amplitude of the Zernike polynomials, was quite different from that encountered in T_2X_. Without correction to T_fw_, the initial error, normalized by |T’_2X_|^2^, was 101% and the product of T’_2X_ and ${\left({\text{T}}_{\text{fw}}^{\text{T}}{\text{T}}_{\text{fw}}\right)}^{-1\left(\text{tik}\right)}$ appeared far from a diagonal matrix, implying low resemblance between T’_2X_ and ${\text{T}}_{\text{fw}}^{\text{T}}{\text{T}}_{\text{fw}}$ Clearly, the random background denotes that the physical misalignment caused undesired interference over all spatial channels. Crucially, the normalized error reduced to 27.7% after correction, which is again close to the experimental benchmark of 15%. Additionally, the resultant product closely resembled the identity matrix, with its integrated on-diagonal energy over the total matrix energy improving from 0.27% to 36.6%. The product of T’_2X_ with its regularized inverse reached 56.8% on-diagonal energy. Therefore, we conclude that T’_2X_ and ${\text{T}’}_{\text{fw}}^{\text{T}}{\text{T}’}_{\text{fw}}$, at least as measured in a single polarization state, are identical to each other, as stated in Eq. (5), which implies that the backward transmission T_bw_ is the same as ${\text{T}}_{\text{fw}}^{\text{T}}$, as described in Eq. (4). This provides evidence of general optical reciprocity and the ensuing transpose symmetry for transmission through a MMF, which serves as a convenient model for general complex media.

### E. Optical phase conjugation based on time reversibility in a reciprocal medium

**FIG. 6.**
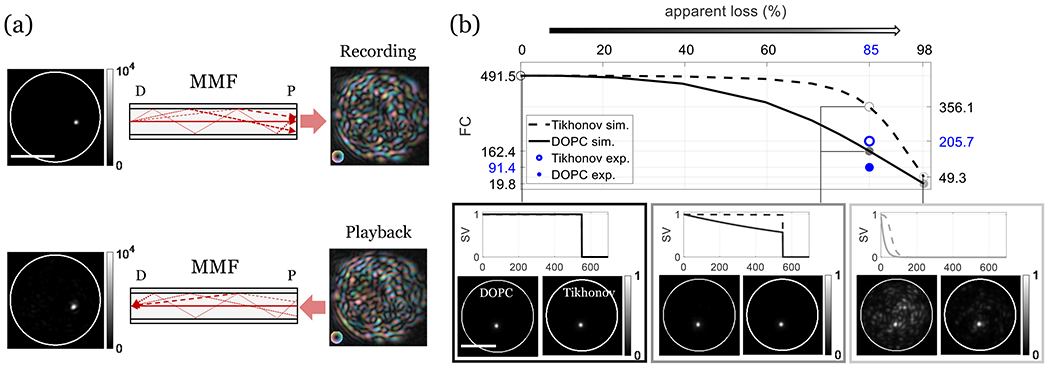
(a) Focusing through a MMF based on time-reversibility with experimental DOPC. D: distal end and P: proximal end. In the recording phase, a laser light is focused on the MMF distal facet and the output speckle field is interferometrically recorded. In the playback phase, the conjugated copy of the recorded wave-front is projected on the proximal facet. This reproduces the distal focal spot, which is clearly visible at the original focusing position and has a FWHM slightly larger due to the limited NA of the MMF. (b) Simulated DOPC- and Tikhonov-inversion-based MMF focusing using f with varying apparent loss. Specific focus examples as well as their corresponding SV distributions (solid curves) are displayed. While the focus remains clearly visible as the background signal increases with loss, Tikhonov regularized inversion compensates to some extent for the mode-dependent loss (dashed curves) and improves the FCs, depending on the loss condition. The white circles outline the fiber facet, and the scale bars are 50 *μ*m.

DOPC based on time reversibility instructs that light propagation can be reversed along its pathway by conjugating its field. In the MMF, a given propagation pathway from the distal input to proximal output can be retraced by a proximal input conjugate to the proximal output, resulting in a distal output in the same spatial channels as the original distal input. This process is represented by substituting $\vec{s}$ in Eq. (1) with the complex conjugate of Eq. (3). Applying Eq. (4), the distal output field (we use $\vec{v}$ rather than $\vec{t}$ to avoid symbol confusion) becomes

(9) $$\vec{v}={\text{T}}_{\text{fw}}{\text{T}}_{\text{fw}}^{\text{†}}{\vec{t}}^{*},$$

where the superscript * indicates the complex conjugation and the superscript † indicates the Hermitian transpose. For a unitary linear system without loss, the Hermitian transpose of T_fw_ equals its true inverse ${\text{T}}_{\text{fw}}^{\text{†}}={\text{T}}_{\text{fw}}^{-1}$ and Eq. (9) reduces to $\vec{v}={\vec{t}}^{*}$. As a result, we can reproduce at the distal end the conjugated wave-front of any initial $\vec{t}$ through the MMF. The experimental realization of Eq. (9) is demonstrated in Fig. 6(a), where we replicated a diffraction-limited focal spot through the MMF with DOPC. The laser, optics, and camera were identical to those in Fig. 2. A 2.5 *μ*m focal spot in the V polarization state was coupled into the MMF through a distal spatial channel. The resulting proximal field was recorded by off-axis holography. We then configured the SLM to send a conjugated copy of the recorded wave-front in the same polarization state back into the MMF from the proximal side. The phase-conjugate light field retraced the forward light propagation and reconstructs the distal focal spot in the V polarization state with a diffraction-limited FWHM of ~4 *μ*m and an FC of 91.4 at the original focusing position. Imperfect FCs (below 550) of the MMF-generated focus can be attributed to losses in the MMF and the measurement system, which violate the power conservation in time reversibility and lead to an only approximate time reversal symmetry in the MMF light transport process. Note that higher FCs of generated foci were obtained with regularized matrix inversion.

To investigate how loss influences the DOPC-based focusing performance, we simulated different loss conditions by replacing the SVs of experimentally measured TMs with exponential functions that have varying decay constants and are multiplied by a step function with a cutoff at 550. A decay constant of zero corresponds to a sharp step function. To include the effect of measurement noise and experimental limitations, we employed two replicate measurements T_fw_ and ${\tilde{\text{T}}}_{\text{fw}}$ of an identical MMF transmission, resulting in different noise realizations in the singular vectors of T_fw_ and ${\tilde{\text{T}}}_{\text{fw}}$. The apparent loss was quantified as the percentage decrease in the square of the Frobenius norm of the TM. For each loss condition, we computed the Hadamard product ${\left({\text{T}}_{\text{fw}}{\tilde{\text{T}}}_{\text{fw}}^{\text{†}}\right)}^{\text{o}2}$, which is the element-wise square of ${\text{T}}_{\text{fw}}{\tilde{\text{T}}}_{\text{fw}}^{\text{†}}$, to simulate MMF focusing and obtain the averaged FC by calculating the averaged ratio of each on-diagonal to the mean value of the corresponding column in the Hadamard product. As shown in the solid curve in Fig. 6(b), the FC declines with increasing loss, resulting in FCs of 491.5, 162.4, and 19.8 at losses of 0%, 85%, and 98%, corresponding to the no loss, equivalent to experimental loss, and substantial loss conditions, respectively. 2D images of example simulated DOPC-based MMF focusing under the conditions were obtained by reshaping a select column of ${\left({\text{T}}_{\text{fw}}{\tilde{\text{T}}}_{\text{fw}}^{\text{†}}\right)}^{\text{o}2}$, revealing a prominent background with high loss. Using, instead, the Tikhonov-regularized matrix inversion approach, as described in Sec. II B, to compute ${\left({\text{T}}_{\text{fw}}{\tilde{\text{T}}}_{\text{fw}}^{-1\left(\text{tik}\right)}\right)}^{\text{o}2}$ improved the MMF focusing and FCs [dashed curve in Fig. 6(b)] of 491.5, 356.1, and 49.3, under the three highlighted conditions, respectively. Figure 6(b) also shows the experimentally achieved FCs of 205.7 from Sec. II B and 91.4 from DOPC here and their apparent loss for comparison. Due to the imperfect wave-front shaping and limited camera dynamic range, the experimental FCs are inferior.

## III. DISCUSSION

Optical reciprocity is a universal principle within linear, nonmagnetic, and static media, even in the presence of loss. It has been previously shown in various formalisms and context^1,2,32,33^ and extends to complex media such as a MMF. We investigated symmetry constraints that reciprocity imposes on bi-directional light transport through a MMF and measured the forward and double-pass TMs, T_fw_ and T_2X_, to demonstrate that ${\text{T}}_{2\text{X}}^{\text{T}}={\text{T}}_{2\text{X}}$ and ${\text{T}}_{2\text{X}}={\text{T}}_{\text{fw}}^{\text{T}}{\text{T}}_{\text{fw}}$. The round-trip transmission reveals a transpose symmetry, and the backward transmission presents a transpose relationship to the forward transmission. Experimentally, we used a single polarization state for illumination and detection, and hence, our measurements correspond only to a subset of the full modes supported by the MMF. Since there is no fundamental difference between spatial and polarization DOFs, we assume this symmetry to also hold for polarization-resolved TMs. We also showed that focusing through a MMF with DOPC, which relies on time reversibility by assuming a loss-free transmission, may have limitation when in practice the transmission suffers from non-negligible loss. This means that reciprocity alone is insufficient for time reversibility, which furthermore requires the absence of losses.

When the MMF is truly loss-free, and we have a fully sampled T_fw_ with uniform SVs, the number of degrees of control is the same as the number of available DOFs. In this case, ${\text{T}}_{\text{fw}}{\text{T}}_{\text{fw}}^{\text{†}}$ is a low-pass filtered identity matrix, suggesting that we can focus through the MMF based on DOPC and achieve an FC close to the number of guided modes. Noise is the only limiting factor, and DOPC- and Tikhonov-inversion based MMF focusing have the same performance. Under our experimental condition, the MMF may leak some of the modes, and only measuring a single polarization state intrinsically eliminates all power in the orthogonal polarization states. Nevertheless, using DOPC, we could still generate a focal spot with an FC of 91.4 through a lossy MMF, treating the transmission as an approximately unitary system, for which ${\text{T}}_{\text{fw}}^{\text{†}}\sim {\text{T}}_{\text{fw}}^{-1}$. On the other hand, experimental focusing through the MMF with regularized TM inversion achieved a better FC of more than 200. This is because the Tikhonov regularization numerically compensates the mode-dependent loss and creates a balanced constructive interference, providing a better focusing performance. However, if the MMF transmission is dissipative, ${\text{T}}_{\text{fw}}{\text{T}}_{\text{fw}}^{\text{†}}$ is far from an identity matrix, and the generated focus with DOPC barely stands out from the speckle background. In this regime, using Tikhonov regularization may only have modest benefit since it only compensates for SVs experiencing modest loss. The DOFs corresponding to SVs smaller than the regularization parameter remain uncontrolled and do not contribute to the constructive interference at intended focus locations.

The implementation of a flexible MMF endoscope remains technically challenging despite recently proposed strategies,^26,34–39^ and the lack of flexibility is the enduring bottleneck for MMF imaging applications. Because the TMs of MMFs are notoriously sensitive to physical fiber deformation,^26^ a flexible MMF endoscope would demand repeated on-site calibration without open distal access in practical endoscopic settings. Imaging through MMFs with certain flexibility based on data-driven approaches has been reported, yet relying on a transmissive regime that requires open distal access.^37,38^ Although calibrating a MMF with only proximal access is a desirable strategy, robust experimental MMF proximal calibration methods remain to be demonstrated. Understanding the reciprocal nature of light propagation through a MMF and the underlying symmetry constraints may help tackle this challenge. In the context of proximal MMF calibration, where the measurement of T_2X_ may be available, the demonstrated symmetry constraint precludes straightforward recovery of T_fw_ or T_bw_, which is needed for imaging through the MMF.^15,26,40,41^ To appreciate this limitation, we can factor T_fw_ into its symmetric and anti-symmetric part based on the second polar decomposition,^42^

(10) $${\text{T}}_{\text{fw}}=\text{AL},$$

where A is orthogonal (A^T^ = A^−1^) and L is transpose symmetric (L^T^ = L). In this case, Eq. (5) becomes

(11) $${\text{T}}_{2\text{X}}={\text{L}}^{\text{2}}.$$

The orthogonal parts cancel each other upon forward and backward transmission, preserving only the symmetric part in the round- trip transmission measurement. Equation (11) states a fundamental restriction: while the symmetric part of T_fw_ can be uniquely retrieved by taking the matrix square-root of the proximally measured T_2X_,^43^ if it has no negative real eigenvalues, the orthogonal part, A, vanishes due to the intrinsic propagation property imposed by the optical reciprocity. Put differently, although a square, complex-valued matrix of dimension *M* has 2*M*^2^ unknown coefficients, the transpose symmetry reduces this number to *M*^2^ + *M*, masking the additional *M*^2^ − *M* of the orthogonal component. This leads to the symmetric degeneracy of T_fw_ even though T_2X_ is known. This explains why T_fw_ cannot be directly retrieved from T_2X_, which complicates strategies for MMF proximal calibration methods.

Previously, Takagi matrix factorization has been proposed to help in recovering T_fw_ from proximal measurements.^36^ A carefully engineered static reflector installed at the distal end of a MMF can provide distinctive reflectivity on individual distal spatial channels, which augments Eq. (5) to

(12) $${\text{T}}_{2\text{X}}={\text{T}}_{\text{fw}}^{\text{T}}{\text{RT}}_{\text{fw}},$$

where R is a real-valued diagonal matrix with sortable on-diagonal elements. By performing Takagi factorization on the accessible T_2X_ and leveraging the transpose symmetry, we have

$${\text{T}}_{\text{2x}}={\text{U}}^{\text{T}}\Sigma \text{U},$$

where U is a unitary matrix and Σ is a real-valued diagonal matrix. If the MMF is loss-free, this would suggest that R = Σ and T_fw_ = DU, where D is an unknown diagonal matrix with entries that are ±1 and might be estimated with prior knowledge. Unfortunately, as shown in Fig. 3, T_fw_ is generally lossy, which compromises this strategy. Based on our findings and arguments, breaking the intrinsic transpose symmetry in the round-trip transmission, installing a calibration element capable of several realizations at the MMF distal end, or introducing new constraints by measuring multispectral round-trip transmission to resolve the degeneracy issue might be the most viable solution toward a flexible MMF endoscope.^6,36,39^ Since reciprocity is ubiquitous, the symmetry principle may also inform non-invasive imaging, coherent wave-control through highly scattering tissues, and electromagnetic communications.

In conclusion, optical reciprocity imposes a symmetry on the bi-directional propagation through a general complex medium regardless of the path complexity or loss. We experimentally demonstrated this symmetry in a looped 1-m-long step-index MMF by measuring the forward and round-trip transmissions. The symmetry prohibits direct retrieval of the forward TM from a round-trip measurement. Thus, MMF endoscopy in a practical setting is fundamentally complicated due to the need to calibrate the MMF without distal access. The insights of light transport within a MMF obtained here may stimulate improved strategies for flexible MMF endoscopy and facilitate efficient sensing and imaging techniques through complex or disordered media.
